# Cognitive Impairment in Diabetes: Rationale and Design Protocol of the Cog-ID Study

**DOI:** 10.2196/resprot.4224

**Published:** 2015-06-09

**Authors:** Paula S Koekkoek, Jolien Janssen, Minke Kooistra, Esther van den Berg, L Jaap Kappelle, Geert Jan Biessels, Guy EHM Rutten

**Affiliations:** ^1^ Julius Center for Health Sciences and Primary Care University Medical Center Utrecht Utrecht Netherlands; ^2^ Department of Neurology Brain Center Rudolf Magnus University Medical Center Utrecht Utrecht Netherlands; ^3^ Experimental Psychology Helmholtz Instituut Utrecht University Utrecht Netherlands

**Keywords:** type 2 diabetes mellitus, cognitive impairment, diagnostic procedure, screening, dementia, elderly

## Abstract

**Background:**

Cognitive impairment frequently co-occurs with type 2 diabetes but is often undiagnosed. Cognitive impairment affects self-management leading to treatment-related complications.

**Objective:**

The aim of this study is to develop a stepped diagnostic procedure, consisting of a screening test complemented by an evaluation by a general practitioner (GP), to detect undiagnosed cognitive impairment in older people with type 2 diabetes.

**Methods:**

The accuracy of two self-administered cognitive tests, the “Test Your Memory” (TYM) and “Self-Administered Gerocognitive Examination” (SAGE) alone, and in combination with an evaluation by a GP will be assessed. A diagnosis of mild cognitive impairment (MCI) or dementia at a memory clinic will serve as reference standard. This cognitive impairment in diabetes (Cog-ID) study will include 513 people from primary care facilities aged ≥70 with type 2 diabetes. The participants will first fill out the TYM and SAGE tests, followed by a standardized GP evaluation for cognitive impairment, including a mini mental state examination (MMSE). Subsequently, participants suspected of cognitive impairment (on either test or the GP assessment) and a random sample of 15% (65/435) of participants without suspected cognitive impairment will be referred to the memory clinic. At the memory clinic, a medical examination, neuropsychological examination, and magnetic resonance imaging (MRI) of the brain will be performed. Participants will also fill out questionnaires assessing health status and depressive symptoms at baseline and after 6 and 24 months.

**Results:**

This research obtained funding and ethical approval. Enrolment started in August, 2012, and all study-related activities will be completed in September, 2016.

**Conclusions:**

With the results from this study, physicians will be able to detect cognitive impairment affecting type 2 diabetes patients through case-finding, and can use tailored care to reduce associated complications. Additionally, the results may stimulate discussions about cognitive impairment and whether early recognition is desirable.

## Introduction

### Background

Patients with type 2 diabetes have an increased risk of cognitive impairment and a doubled risk of dementia compared to people without diabetes [1,2]. Cognitive impairment often remains unrecognized by physicians, even when patients or their relatives express complaints [3,4]. This is an important problem since in patients with type 2 diabetes, cognitive impairment is associated with impaired self-management and an increased incidence of diabetes-related complications [5,6]. Early recognition of cognitive impairment could assist the general practitioner (GP) in taking appropriate, personalized measures in diabetes management to prevent complications [7].

Routine screening for cognitive impairment in elderly patients with type 2 diabetes has been advocated [8]. The American Diabetes Association advises to individualize diabetes treatment and to adjust management to the preserved capacity of patients, thereby specifically taking into account cognitive functioning [9]. However, compared with other potential complications and co-morbid conditions of type 2 diabetes, the diagnostic evaluation of diabetes-associated cognitive impairment is underdeveloped. While screening algorithms have been established for microvascular complications, such as retinopathy or nephropathy, there is no established method to detect undiagnosed cognitive impairment. The ideal procedure for the assessment of possible disturbances of cognitive functioning should be easy and quick to perform. The procedure should readily identify people who require further, more elaborate and time consuming, evaluations by the GP or possibly referral to a memory clinic. Unfortunately, administration of most cognitive tests already requires a lot of time from a physician, nurse, or other health care worker. In addition, currently available tests with the shortest administration times tend to cover only certain aspects of cognition, particularly those affected in Alzheimer’s disease. Moreover, these tests are much less accurate in identifying people with other conditions, in particular vascular cognitive impairment [10].

These issues may be resolved by the recent introduction of self-administered cognitive tests, such as the Test Your Memory (TYM) [11] and the Self-Administered Gerocognitive Examination (SAGE) [12] tests. In a memory clinic setting, these tests have been shown to measure a broader range of cognitive domains than the mini mental state examination (MMSE) and they were also able to detect mild cognitive impairment (MCI) [11-13]. Therefore, in our view, these self-administered cognitive tests could be promising tools for the detection of cognitive impairment in type 2 diabetes in primary care.

The ultimate goal of a diagnostic procedure for cognitive impairment is to improve clinical outcomes and patients' quality of life. However, before the effect of a diagnostic procedure can be evaluated, which specific tests to include must be determined. The latter is examined in this cognitive impairment in diabetes (Cog-ID) study. Here, we aim to establish a reliable, valid, and efficient stepped diagnostic procedure to detect cognitive impairment in patients ≥70 years of age with type 2 diabetes, starting with the TYM and the SAGE tests. It is unknown which of these two tests is best suited for application in a primary care setting; therefore we will assess the accuracy and feasibility of both. In addition, we will describe how early detection of cognitive impairment affects treatment and quality of life in an observational study that is part of the main study. Together, the results will help shape future studies with the goal of answering the unresolved, but increasingly relevant and heavily debated question [14], whether early recognition of cognitive impairment in patients with type 2 diabetes will help the GP to take appropriate measures in disease management, and ultimately prevent treatment-related complications. Future studies are needed to assess the effect of the established diagnostic procedure on clinical outcomes in a randomized controlled trial.

### Objectives

Our overall aim is to establish a reliable, valid, and efficient stepped diagnostic procedure to detect undiagnosed cognitive impairment in patients ≥70 years of age with type 2 diabetes. The procedure will consist of a self-administered cognitive test and an evaluation by a GP. Additionally, we will describe how early detection of cognitive impairment affects treatment and quality of life in participating patients in a parallel observational study. The specific objectives of the study are (1) to assess the validity of two self-administered cognitive tests (TYM and SAGE) in detecting undiagnosed cognitive impairment in elderly patients with type 2 diabetes in a primary care setting and to select the best instrument, (2) to assess the diagnostic accuracy of a standardized evaluation by a GP in detecting undiagnosed cognitive impairment in patients with type 2 diabetes, (3) to estimate the accuracy and efficiency of the best cognitive test(s) combined with the evaluation by the GP, and (4) to describe the effect of the diagnostic procedure on several aspects of diabetes care (ie, treatment targets and appointment schedules) and patients’ quality of life.

## Methods

### Study Participants

General practitioners (GPs) in the surroundings of Utrecht, the Netherlands, will be asked to select patients with type 2 diabetes mellitus ≥70 years of age. Exclusion criteria include a diagnosis of dementia, previous investigation at a memory clinic, and the inability to write or read in Dutch. Patients with a disorder that might influence cognitive functioning, like substance abuse or a psychiatric or neurological disorder, but without a diagnosis of cognitive impairment are not excluded as we are interested in the presence of unknown cognitive impairment regardless of the cause. Eligible patients will receive a letter from their GP with information regarding the study. Patients will be asked to return the response form on which they can mark whether or not they are willing to participate. In the case of non-response, one reminder will be sent.

### Screening Tests

#### Test Your Memory Test

The TYM is developed to test a range of cognitive functions and consists of 10 tasks [11]. It is a self-administered test and takes a patient around 5 minutes to complete. The tasks include orientation (10 points), ability to copy a sentence (2 points), semantic knowledge (3 points), calculation (4 points), verbal fluency (4 points), similarities (4 points), naming (5 points), visuospatial abilities (2 tasks, total 7 points), and recall of a copied sentence (6 points). The ability to complete the test without help is an 11^th^ task (5 points); because of our study design all patients will receive these 5 points. The maximum score is 50 points. A score of ≤39 is suggestive of dementia [11]. The TYM was translated into Dutch and then translated back to English by a bilingual native English speaker, which resulted in a version almost identical to the original.

#### Self-Administered Gerocognitive Examination

The SAGE measures cognitive functioning in the domains of orientation (4 points), language (4 points), memory (2 points), executive function (4 points), calculations (2 points), abstraction (2 points), and visuospatial abilities (4 points) [12]. Furthermore, the SAGE includes several questions on demographic information, medical and family history, and current status. The maximum score is 22 points. A score of ≤14 is suggestive of dementia [12]. Like the TYM, the SAGE was translated into Dutch and then back into English, which resulted in a version almost identical to the original.

### The Diagnostic Strategy

#### Part 1: Home Visit

Participants will be visited at home by a research physician (a trainee GP). The home visit will take about 1 hour. The participant will be asked to fill out the TYM, SAGE, and a questionnaire assessing health status and depressive symptoms, including the Short Form Health Survey (SF-36)[15], EuroQol (EQ)-5D and EQ-VAS [16], and the Center for Epidemiologic Studies Depression Scale (CES-D) [17]. The research physician will be blinded for the scores on the TYM and the SAGE, and will not provide any assistance in filling out the questionnaires. Following the questionnaires, the research physician will administer a standardized diagnostic interview based on the Dutch guideline for case finding of dementia by GPs to both the participant and (if possible) a close informant [18], representing the evaluation by the GP. The interview will include demographic variables, educational level, and living conditions, as well as a medical history and a list of cognitive complaints (Table 1). After the interview, the MMSE will be administered. The MMSE consists of 11 tasks including the domains orientation in time (5 points), orientation in space (5 points), registration of three words (3 points), concentration and calculation (5 points), recall of three words (3 points), language (8 points) and visuospatial abilities (1 point). The maximum score is 30 points with a higher score indicating a higher level of cognitive functioning. A score of ≤24 is suggestive of dementia.

Based on the history taken, the research physician will decide whether the participant should be classified as "suspected of cognitive impairment" or "no cognitive impairment" according to the criteria for MCI and dementia [19,20]. If the MMSE score is ≤24, the participant will always be classified as "suspected of cognitive impairment".

**Table 1:** List of questions about acquired cognitive symptoms for the participant and informant.

Questions		Patient^a^	Informant^a^
Do you have memory problems?			
Do other people think you are forgetful?			
Do you forget names of relatives or peers?			
Do you often lose things?			
Do you have to write more things down to remember it than you were used to?			
Are there activities you stopped doing in the past five years (and why)?			
Do you visit friends or family less often?			
How does cooking, grocery shopping and the household go?			
Do you have trouble managing your finances?			
Do you have trouble driving a car or using public transport?			
Do you need help getting dressed?			
Do you sometimes forget what month or year it is?			
Can you independently manage your medication?			
Can you follow the news in the paper or on television?			
Do you have problems with walking or holding your balance?			
Did you lose weight unintentionally in the past years?			
Has your smell or taste changed in the past years?			
Are you depressive?			
Can you still have pleasure in things?			
Do you have problems with hearing or vision?			
**The following 3 questions to be completed by the informant**			
	Do you think his/her personality has changed?		
	Did you take over tasks from the participant (and why)?		
	Does he/she repeat things often?		
**Observational points**		**Yes**	**No**
	Inability to find the correct words		
	Many repetitions or hesitations		
	Often does not understand the question		
	Head turning sign		
	Inconsistencies or confabulation		
	Poor grooming		

^a^input fields to be filled in with the answers

#### Part 2: Selection Criteria for Memory Clinic Visit

**Figure 1:**
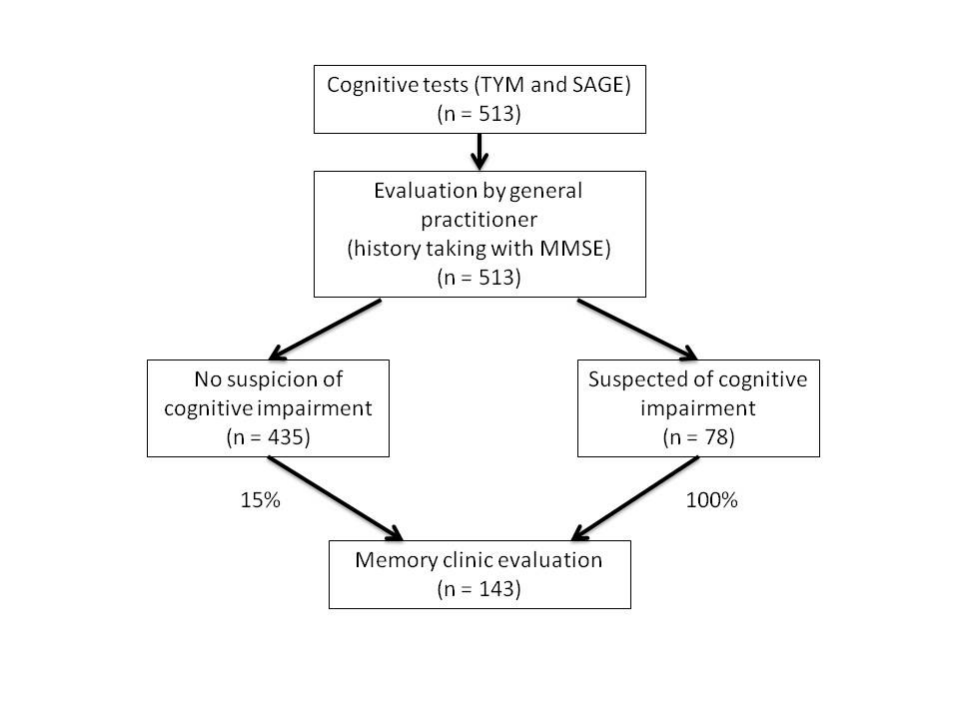
Study flowchart.

After the home visit, an independent physician, not involved in the home visit or in the memory clinic, will determine whether the participant will be selected for a visit to the memory clinic of the University Medical Centre Utrecht. To minimize the influence of the increasing experience of the research physician because of the growing number of home visits during the study period, the research physician who visited the participant at home will not be informed about the results of the memory clinic. The following 3 criteria will be used to decide whether a participant will be invited to the memory clinic (1) a classification of “suspected of cognitive impairment” by the research physician, (2) a score of ≤39 on the TYM, and (3) a score of ≤14 on the SAGE. When a participant scores positive on one of these criteria, the participant will be invited to the memory clinic. In addition, a random sample of 15% (65/435) of participants with negative scores on all 3 criteria will be invited to the memory clinic (see sample calculation below and Figure 1).

#### Part 3: Memory Clinic Visit

All professionals involved in the memory clinic will be blinded to the results of the TYM and SAGE. The visit to the memory clinic will take half a day and will consist of a standardized memory clinic workup.

### Medical Examination

Participants will be examined by a (trainee) neurologist who will perform a diagnostic interview and a neurological examination, administer the Cambridge Cognitive Examination (CAMCOG) [21], and measure body weight, height, and blood pressure. Body mass index (BMI) will also be calculated. In addition, the Disability Assessment for Dementia (DAD) [22] and the Neuropsychiatric Inventory (NPI) [23] will be administered to a caregiver to measure functional abilities of daily living and to assess the presence of neuropsychiatric symptoms.

### Neuropsychological Assessment

A neuropsychologist will administer a 90-minute standardized neuropsychological assessment examining memory, information processing speed, attention and executive functioning, and visuoconstruction. The division in cognitive domains will be made a priori, according to standard neuropsychological practice and cognitive theory [24]. The domain "memory" will be assessed by the subtest Digit Span of the Wechsler Adult Intelligence Scale -Third edition (WAIS-III) , the Rey Auditory Verbal Learning Test (RAVL), and the delayed recall of the Rey-Osterrieth Complex Figure Test (ROCF). The domain "information processing speed" will be assessed by the trail-making test (part A), the Stroop Color-Word Test (parts 1 and 2), and the subtest symbol digit substitution of the WAIS-III. The domain "attention and executive function" will be assessed by the trail-making test (part B; ratio score), the Stroop color-word test (part 3; ratio score), the visual elevator test, a letter fluency test using the letters ‘N’ and ‘A’, and category fluency (animal naming). The domain "visuoconstruction" will be assessed by the copy trial of the ROCF, the Judgment of Line Orientation (JLO), and the *Visual Object and Space Perception Battery* (VOSP). Furthermore, the premorbid level of intelligence (intelligence quotient (IQ)) will be estimated by the Dutch version of the National Adult Reading Test (NART). Educational level will be recorded in seven categories and subsequently translated into years of education. Frailty will be examined with the Short Physical Performance Battery (SPPB).

### Additional Examinations

MRI data will be acquired on a Philips 3.0 Tesla scanner using a standardized protocol and consisting of a T2-weighted scan (48 continuous slices, reconstructed voxel size: 0.99 × 0.99 × 3.00 mm^3^), a 3D T1 scan (192 continuous slices, reconstructed voxel size: 1.00 × 1.00 × 1.00 mm^3^), a fluid attenuated inversion recovery (FLAIR) scan (48 continuous slices, reconstructed voxel size: 0.96 × 0.95 × 3mm^3^), and diffusion-weighted MRI data using a single-shot spin echo planar imaging sequence (48 contiguous slices, acquired isotropic voxel size 2.50 mm, 45 isotropically distributed diffusion-sensitizing gradients with a b value of 1200 s/mm^2^, and one b=0 s/mm^2^).

Venous blood samples will be drawn to determine non-fasting blood glucose, HbA1c, blood count, lipid-levels (HDL, LDL, total cholesterol, triglycerides), thyroid function, liver functions, and kidney function.

### Cognitive Impairment Diagnosis

Within two weeks of the visit to the memory clinic, a multidisciplinary team meeting will be planned with a neurologist, the neurology resident, and the neuropsychologist to establish the diagnosis. Cognitive impairment ( ie, MCI or dementia) is our primary outcome. For the diagnosis of dementia, the DSM-IV criteria will be used [19]. In short, dementia will be defined as memory impairment and impairment in at least one other cognitive domain, including aphasia, apraxia, agnosia, and executive functioning, that significantly affects social or occupational functioning compared to the previous level of functioning, and that is not caused by a delirium. MCI will be diagnosed according to the criteria by Winblad et al, and defined as not normal, not demented, with cognitive complaints that can be objectified by a neuropsychological assessment and/or evidence of decline over time, and preserved basic activities of daily living [20]. In addition, the presumed etiology of dementia will be specified (eg, Alzheimer’s disease).

Guided by the diagnosis, tailored treatment advice will be given to the participants’ GP regarding management of the diabetes treatment and cognitive impairment. Advice for the diabetes treatment will consist of re-evaluation of the proper glycemic target and the risk of insulin treatment. As well, advice evaluating the need for extra support for participants unable to meet treatment goals or in need of tools, for example a memory aid for appointments or medication, will be provided.

### After the Diagnosis

The results of the visit to the memory clinic and the treatment advice will be sent to the GPs who will discuss the results with the participant. Subsequently, the GP and the participant will decide together what actions will be taken. Further support by the memory clinic will be available if considered desirable by the GP and the participant.

### Follow-Up

Following the home visit (6 months), participants will receive a follow-up questionnaire, including the SF-36, EQ-5D, EQ-VAS, and the CES-D to evaluate the course of their health status, quality of life, and depressive symptoms. A questionnaire asking whether and how many hypoglycemic events, visits to emergency services, and hospital admissions they experienced will also be included. In addition, participants will be asked whether they regret their participation in the study and whether they would again participate in the study. A second follow-up questionnaire with the same questions will be sent after 24 months.

After the home visit (6 months), the medical records of the participants will be examined to obtain information on the medical history, values of recent diabetes controls (HbA1c, lipids, creatinine, weight, height, blood pressure), complications (hypo- or hyperglycemic events), and visits to emergency services and hospital admissions in the year before and six months after participation in the study.

To further assess the impact of the study on participants’ treatment, GPs of participants that attended the memory clinic will receive a questionnaire 6 months after the evaluation at the memory clinic to assess whether the study led to new insights and whether it changed their treatment plan (Textbox 1).

**Follow-up questions for the general practitioner (GP).**

1. Did the result come as a surprise to you or did you expect it? And why?
2. Do you agree with the result of the memory clinic? And why?
3. Did you adjust your diabetes treatment or management because of the results? And why?
4. Did the results have consequences for your overall medical treatment of the patient? And why?

### Statistical Analysis

The diagnosis of cognitive impairment (MCI or dementia) at the memory clinic will be used as the reference standard. To address the first two objectives, participants will be classified as true positive, false positive, false negative, or true negative separately for the evaluation by the GP, TYM, and SAGE.

Not all of the patients in our study will receive the reference standard, which could lead to partial verification bias [25]. However, if only patients with the reference standard were included in the analysis (complete case analyses), the results would be biased because the selection of the patients with the reference standard will not be at random [25]. A reliable method to reduce this bias is to impute the reference standard [25]. A cognitive impairment diagnosis (yes or no) in the memory clinic will, therefore, be imputed for patients who did not attend the memory clinic. Imputed databases (N=10) will be generated with the predictors TYM, SAGE, MMSE, GP evaluation, as well as age, gender, educational level, living situation, and score on the domain mobility of the EQ-5D. The latter two are chosen because they can influence why some patients did not attend the memory clinic. With these imputed numbers, the sensitivity, specificity, positive predictive value (PPV), and negative predictive value (NPV) will be calculated.

The extent to which the cognitive tests and the evaluation by the GP discriminate between participants with and without cognitive impairment will be determined by the area under the receiver operating characteristic (ROC) curve. Next, the optimal cutoff values of the tests for this population will be determined according to the best combination of corresponding sensitivity and specificity assessed with the Youden index. The Youden index measures the effectiveness of a diagnostic marker and enables the selection of an optimal cutoff point [26]. By means of the ROC curve and the best combination of diagnostic values, the optimal instrument will be selected.

For assessing the accuracy and efficiency of the diagnostic procedure (ie, the cognitive test combined with history taking; objective 3) the results of the best cognitive test and the evaluation by the GP will be combined. This should reflect the future implementation of the stepped diagnostic procedure, in which a GP will only evaluate those patients with a positive test result. Participants will be categorized in the “test positive” group when both the best cognitive test and the evaluation by the GP are positive. This combination will likely have a higher PPV than the cognitive test or the evaluation by the GP alone, leading to a more efficient diagnostic procedure. The added value of the GP’s evaluation will be assessed by calculating the adjusted ROC curve and the net reclassification index [27].

The fourth objective of this study will be addressed by comparing the difference in health status and depressive symptoms between those with and without a diagnosis of cognitive impairment, both at baseline and at the 6- and 24-month follow-up, taking into account potential baseline differences of relevant parameters. In addition, we will describe the changes that were made in diabetes care by comparing the diabetes management before and after study participation (ie, changes in treatment, number of hypo- or hyperglycemic events, emergency and hospital visits).

### Sample Size Calculation

For our sample size calculations, we assumed a prevalence of undiagnosed cognitive impairment of 8%. Since little quantitative information is available on the prevalence of undiagnosed cognitive impairment, we based this assumption on four considerations. The first assumption is the prevalence of dementia in the Dutch population >65 years of age is around 16% [28].The prevalence of cognitive impairment will be even higher if MCI is also considered. The second is that around half of all patients with cognitive impairment are undiagnosed. The third is the prevalence of cognitive impairment is higher in people with diabetes. And the fourth is the oldest old, in whom dementia prevalence is highest, are least likely to participate in research projects.

In previous research in adults aged ≥59 years recruited from geriatric and memory clinics and facilities for seniors, the SAGE had a PPV of 64%, a NPV of 95%, a sensitivity of 79%, and a specificity of 95% with regard to diagnosing cognitive impairment [12]. In a memory clinic population, the TYM had a specificity of 95%, a sensitivity of 81%, a PPV of 64%, and a NPV of 98% at a cutoff score of 39 points for Alzheimer's disease. In our view, a new cognitive test should have a PPV comparable with that of the most commonly used instrument, the MMSE, which has a PPV of 53.6% for the diagnosis of dementia in primary care [29]. Therefore, for our sample size calculation, we set the lower margin for the estimated PPV at 53% (ie, 11% below the previously established PPV of 64%). With this margin and an alpha of 5% and one-sided testing (we are only interested in the lowest 5% of cognitive scores), 52 participants with a positive test result (0.11= 1.65*√(0.64*(1-0.64)/n)) are needed to have reliable, interpretable results. To achieve this number of test positive participants, given an assumed prevalence of 8% and a sensitivity of 79%, 513 participants are required. Given the test features of the TYM, this sample size should also be sufficient to determine the accuracy of the TYM. As participants will be referred to the memory clinic based on the results of all 3 tests (TYM, SAGE, and evaluation by the GP), and the results of the tests will probably not completely overlap, the group "suspected of cognitive impairment" will be larger than the group that will be tested positive on the SAGE alone. We estimate that the former group will be 50% larger than the SAGE-positive group (ie, 78 people are estimated to be in the group “suspected of cognitive impairment”). All these 78 participants will be invited to attend the memory clinic in order to establish the true and false positive rates of each of the tests. In addition, a sample (14.9%, 65/435) of the participants in which all 3 tests are negative (the screen-negatives) will be invited to the memory clinic to establish the true and false negative rates of each test. Hence, 143 participants in total will be evaluated at the memory clinic (Figure 1).

Because of uncertainty on the actual prevalence of undiagnosed cognitive impairment in our cohort, an interim analysis is planned after the inclusion of 80 participants. During this interim analysis, only the proportion of participants classified as "suspected of cognitive impairment" will be checked without unblinding the test scores or the findings at the memory clinic. If the proportion deviates significantly from our assumptions we will adjust the sample size of the study population accordingly.

### Regulation Statement

This study will be conducted according to the principles of the declaration of Helsinki and in accordance with the Dutch law on Medical Research Involving Human Subjects Act (WMO).

### Ethics Committee Approval

The cognitive impairment in diabetes (Cog-ID) study was approved by the medical ethics committee of the University Medical Centre Utrecht, the Netherlands. Written informed consent will be obtained from all participants.

## Results

Funding was obtained through the EFSD/Lilly Mental Health and Diabetes Program in 2012. Participant enrolment started in August, 2012. All study-related activities will be completed in September, 2016. The first results are expected to be published in 2015.

## Discussion

This cognitive impairment in diabetes (Cog-ID) study will provide a stepped diagnostic procedure to identify patients with type 2 diabetes and undiagnosed cognitive impairment, which can be readily implemented in daily practice. This is essential to improve the care for this vulnerable patient group. We will have information on the diagnostic accuracy of two new cognitive tests, the TYM and the SAGE, and whether these tests can be used in a diagnostic procedure (ie, combining a cognitive test with history taking by a GP) to detect cognitive impairment in primary care. In addition, we will collect observational data on the impact of such diagnostic procedures on several aspects of patients’ lives (health status, depressive symptoms, complications, and diabetes treatment) after 6 and 24 months. Physicians often assume that informing the patient about a diagnosis of cognitive impairment will negatively influence their health status, quality of life, and depressive symptoms [30]. However, one could also argue that undiagnosed cognitive impairment might cause a reduced quality of life and depressive symptoms, because it is likely to impact patients. If these aspects of patients' lives are affected by undiagnosed cognitive impairment, and could be ameliorated by informing the patient, then the tailoring and possibly the adjustment of treatment and/or organizing support could be another argument as to the importance of detecting cognitive impairment at an early stage.

A potential bias in diagnostic studies in which not all patients receive the reference standard is partial verification bias [25]. However, we will try to reduce this verification bias by imputing the reference standard in participants that do not visit the memory clinic. This method has been shown to give reliable estimates of missing reference data [25].

With the information from this study, we can advise GPs on how to assess cognitive functioning in their patients so they can adjust diabetes treatment to the preserved capacities of their patients, as advocated by the American Diabetes Association, and consequently might prevent treatment-related complications. In addition, the results will form a base for future discussions on whether the early recognition of cognitive impairment in patients with type 2 diabetes with a case-finding strategy is desirable.

## Appendix

Multimedia Appendix 1 Confirmation of grant approval.

Multimedia Appendix 2 Confirmation of ethical approval.

## Abbreviations

CES-D: Center for epidemiologic studies depression scale
Cog-ID: Cognitive impairment in diabetes study
EQ-5D: EuroQol 5 dimensions
EQ-VAS: EuroQol visual analogue scale
GP: General practitioner
MCI: Mild cognitive impairment
MMSE: Mini-mental state examination
NPV: Negative predictive value
PPV: Positive predictive value
ROC: Receiver operating characteristic
SAGE: Self-Administered Gerocognitive Examination
SF-36: Short form health survey 36
TYM: Test Your Memory test
