# Supplementary material for: Cognitive Impairment in Diabetes: Rationale and Design Protocol of the Cog-ID Study
Source: JMIR Res Protoc. 2015 Jun 9;4(2):e69. doi: 10.2196/resprot.4224 (PMC4526928; doi:10.2196/resprot.4224)
Supplement: Multimedia Appendix 2 [file resprot_v4i2e69_app2.pdf]

Universitair Medisch Centrum
Utrecht

Medical Research Ethics Committee

University Medical Center
Division Julius Centre for Health Sciences and Primary Care
t.a.v. Drs. P.S. Koekkoek
Internal mail : STR.6.131

Mrs. M. van de Loo – Waller
Telephone ++31 0887556376
Internal mail D 01.343
E-mail metc@umcutrecht.nl
www.umcutrecht.nl/metc

Date
21 June 2012

Our reference
MvdL/ck/12/022280

Your reference

Subject
METC protocol number 11-462/O
WMO-Approval mono-center

Dear Ms. Koekkoek,

In her meeting of 22 May 2012 the Medical Research Ethics Committee (METC), accredited on 11 November 1999 by ex section 16 of the WMO[1], has reviewed the research protocol number 11/462, entitled **"Screening for cognitive impairment in patients with type 2 diabetes"**, submitted by Prof. Dr. G.E.H.M. Rutten from Utrecht and commissioned by University Medical Center Utrecht.

The committee has considered the suitability of the research proposal, the objectives of and the rationale for the research proposal, the study design, the ethical aspects, the methods and the materials that will be used to obtain and document the informed consent of the research subjects. The committee, operating in accordance with the WMO, ICH-GCP guidelines and other relevant Dutch and/or European laws, has judged that the rights, the safety and the well-being of the subjects in the research proposal are protected and, within its competence in accordance with section 2, second subsection sub a, of the WMO, reviewing section 3 of the WMO, approves the execution of this research proposal.

The committee declares that:

- the expected scientific results of the research justify the burden for the research subjects;
- the trial will not be carried out in conflict with WMO section 4, 1st subsection, section 5 and section 6, 1st subsection;
- the research subjects will be appropriately informed (WMO section 6, subsection 3 to 7 inclusive).
- The insurance, in accordance with the provisions of the WMO and the Medical Research (Human Subjects) Compulsory Insurance Decree of 23 June 2003, is adequately arranged.

In addition, the committee wishes to inform you that as of 1 March 2006, parties responsible for the conduct of the study should be covered by liability insurance before commencement of the study. Existing liability insurance will suffice.

[1] Medical Research (Human Subjects) Act

3006591

Please pay special attention to the following:
- you are required to inform the committee (by e-mail if you wish) of the date of inclusion of the first subject in the study;
- after one year, you are required to submit a progress report of the study and yearly thereafter. Progress report forms may be downloaded from the METC website: (www.umcutrecht.nl/metc);
- **you are also required to submit a progress report after 150 research subjects have been included in the study**
- you are required to inform the committee of the date the study ends (the date which the last research subject undergoes the last procedure connected to the trial);
- a final report of the study, as well as any publications or abstracts resulting from the study, should be submitted to the committee within one year of completion of this study;
- this approval looses its validity when the study has not started within one year after this decree.

You are required to inform the committee immediately:
- in case the study is prematurely terminated. The principle investigator or sponsor where appropriate, should inform the committee the reason for the termination of the study;
- in case of a serious unexpected side effect or an unexpected event, or when the study progresses in an otherwise unforeseen manner (WMO article 10). For regulations regarding the reporting of SUSAR's and SAE's to the committee, please see www.ccmo.nl;
- any new information with unfavourable consequences for the safety of the research subjects or the conduct of the clinical trial.

Any change in the protocol, however small, should be submitted to the METC for approval.

Finally, the committee wishes to inform you that the study may only be started after approval from the board of directors of your institution. The letter of approval from de Board of Directors of the UMC Utrecht to start the study in the UMC Utrecht usually follows the receipt of the committee approval promptly.

Under section 7.1 of the General Administrative Law Act a party whose interest is directly related to this decision may lodge an administrative appeal with the Central Committee on Research Involving Human Subjects (CCMO) within six weeks after this decision's date. An administrative appeal has to be addressed to: CCMO, PO box 16302, 2500 BH The Hague, The Netherlands.

With kind regards,
on behalf of the METC,

M. D. van de Loo – Waller

M. van de Loo – Waller

A committee composition list and the committee regulations can be withdrawn from the METC office.
This is an English translation of the Dutch Approval letter for this study.
For a complete list of the documents the committee has approved see the formal Dutch Approval letter (with reference number: MvdL/ck/12/019198).
