# Supplementary material for: Cognitive Impairment in Diabetes: Rationale and Design Protocol of the Cog-ID Study
Source: JMIR Res Protoc. 2015 Jun 9;4(2):e69. doi: 10.2196/resprot.4224 (PMC4526928; doi:10.2196/resprot.4224)
Supplement: Multimedia Appendix 1 [file resprot_v4i2e69_app1.pdf]

EFSD

European Foundation for the Study of Diabetes

Dr Geert Biessels
University Medical Center Utrecht
Rudolf Magnus Institute of Neuroscience
Department of Neurology, G03.228
PO Box 85500
3508 GA Utrecht
The Netherlands

BY POST & EMAIL

09 February 2012

Dear Dr Biessels

EFSD/Lilly Mental Health and Diabetes Programme
Screening for cognitive impairment in patients with type 2 diabetes (SCIDS)

On behalf of the European Foundation for the Study of Diabetes and the Programme Partner Lilly, we have the pleasure of informing you that based on the decisions of the Review Committee, your grant application has been accepted for funding in the sum of Euro 200,000 (two hundred thousand). This sum will be administered in equal installments over two years. Please note that you are expected to provide an annual interim progress report in order to receive further funding installment/s.

Grant activation documentation is attached to the postal version of this correspondence for completion and return in due course. In the meantime, we would be pleased to receive a brief e.mail from you confirming that you accept the funding amount as stated above.

If you have any questions, please do not hesitate to contact the EFSD Office, and may we take this opportunity to wish you every success with your research project.

Yours sincerely

Dr Viktor Jörgens
Executive Director - EFSD

Dr Monika Grüsser
Vice Director – EFSD

President: Prof A J M Boulton – UK
Vice President: Prof F Bosch – Spain
Vice President: Prof S Del Prato – Italy
Honorary Secretary: Prof M Walker – UK
Honorary Treasurer: Prof M Roden – Germany
Chair – PGEC: Prof C J Tack – Netherlands
Editor-in-Chief – Diabetologia: Prof J Zierath - Sweden

Office:
Executive Director: Dr V Jörgens
Vice Director: Dr M Grüsser

Chairman:
Prof P A Halban – Switzerland

Rheindorfer Weg 3
40591 Düsseldorf, Germany
Tel: +49-211-758469-0
Fax: +49-211-758469-29
Foundation@easd.org
www.EuropeanDiabetesFoundation.org
